# Navigating resilience: Mental health self-care among middle-aged and older LGBTQ+ adults in Thailand

**DOI:** 10.1371/journal.pone.0353372

**Published:** 2026-07-17

**Authors:** Nanchatsan Sakunpong, Ramida Mahantamak, Chawitra Tantimala, Kitsiri Chochiang, Sarah Abboud

**Affiliations:** 1 Behavioral Science Research Institute, Srinakharinwirot University, Bangkok, Thailand; 2 Department of Education, Faculty of Social Sciences and Humanities, Mahidol University, Nakhon Pathom, Thailand; 3 Faculty of Humanities and Social Sciences, Chandrakasem Rajabhat University, Bangkok, Thailand; 4 Faculty of Science, Prince of Songkla University, Songkhla, Thailand; 5 University of Illinois Chicago, College of Nursing, Chicago, Illinois, United States of America; Shandong University, CHINA

## Abstract

**Introduction:**

This study explores how middle-aged and older LGBTQ+ adults in Thailand engage in mental health self-care within complex social and cultural environments. While global research has largely reflected Western perspectives, little is known about how self-care is practiced in non-Western, Buddhist, and collectivist contexts. This study situates mental health self-care as both an individual and socially embedded process shaped by cultural beliefs, kinship norms, and structural inequalities.

**Methods:**

A qualitative study was conducted with 30 LGBTQ+ participants aged 50–68 from Thailand, using thematic analysis. Semi-structured, in-depth interviews were carried out online and analyzed within a thematic framework to identify recurring patterns and culturally grounded meanings.

**Results:**

Two overarching themes emerged: (1) Socio-cultural backgrounds shaping mental health self-care, which included Family and Childhood Memories, Community Environment, Cultural Beliefs and Mental Health, and Intimacy and Romantic Experience; and (2) Lived Experiences of Mental Health Self-Care, encompassing strategies for Coping with Stigma and Discrimination, Barriers to Mental Health Services, Cultivating Joy, Pride, and Self-Reliance, Community Contribution and Advocacy, Physical Health as Mental Anchor, and Self-Education on Mental Health. Self-care was intertwined with Buddhist ethics, caregiving norms, and digital peer support, reflecting both adaptation and quiet resistance within Thai society.

**Conclusion:**

Mental health self-care among older LGBTQ+ Thais represents a culturally informed resilience that bridges individual agency and collective well-being. The findings highlight the importance of integrating social determinants of health and cultural context into inclusive mental health policies and community-based interventions for aging LGBTQ+ populations.

## Introduction

Lesbian, gay, bisexual, transgender, queer (LGBTQ+) and other gender and sexual minority individuals are at elevated risk of mental health problems. This vulnerability may be particularly pronounced among middle-aged and older adults. A growing body of evidence indicates that LGBTQ+ individuals experience higher rates of depression, anxiety, loneliness, and suicidality compared to their heterosexual and cisgender peers [1,2]. These disparities often persist or intensify with age, compounded by cumulative experiences of minority stress, discrimination, social invisibility, and inadequate access to affirming mental health services [3–6]. Yet, despite these risks, aging LGBTQ+ populations remain underrepresented in public health research, particularly in non-Western and middle-income contexts.

Mental health self-care is increasingly recognized as a critical aspect of psychological well-being, particularly for marginalized populations such as LGBTQ+ individuals who face disproportionate exposure to stress and discrimination. In this study, mental health self-care is defined as a set of intentional and proactive strategies used to manage psychological distress, enhance emotional regulation, and sustain mental wellness in everyday life [7,8]. These strategies are grounded in a holistic understanding of mental health, which includes biological, psychological, social, and environmental determinants [9]. Common self-care approaches include identity affirmation, mindfulness practices, boundary setting, and engagement with affirming communities. For LGBTQ+ individuals, such practices function not only as coping mechanisms but also as tools for resisting systemic oppression, social invisibility, and chronic minority stress. This is especially important for those experiencing intersecting vulnerabilities, such as older age, gender nonconformity, low socioeconomic status, or chronic health conditions [3,10,11]. Among middle-aged and older LGBTQ+ adults, protective factors such as self-acceptance, emotion regulation, and access to affirming social networks have been shown to support effective mental health self-care and promote psychological resilience. These strategies are particularly critical in navigating chronic minority stress and gender-related challenges across the life course [10,12]. These factors, however, vary across national and cultural contexts, underscoring the importance of examining self-care within the socio-cultural environments that shape individual and community resilience.

However, the presence, meaning, and effectiveness of self-care strategies vary considerably across countries and are deeply shaped by local socio-cultural environments. In high-income countries with inclusive health policies and visible LGBTQ+ infrastructures, such as the United States and parts of Europe, access to affirming mental health services, legal protections, and supportive aging programs has been associated with greater engagement in self-care, reduced psychological distress, and enhanced resilience among LGBTQ+ older adults [13–15]. In contrast, in many parts of Southeast Asia and Latin America, where formal legal protections for LGBTQ+ individuals may be limited or inconsistently enforced, community-based coping strategies, such as chosen families and grassroots health initiatives, frequently function as vital sources of resilience and psychological support [11,16–18]. These global variations highlight that mental health self-care is not a universal construct but one that is profoundly shaped by the intersections of culture, policy, religion, and social norms.

In the Thai context, specific socio-cultural dynamics present both opportunities and constraints for mental health self-care among LGBTQ+ adults. Buddhist values such as compassion, forgiveness, and non-violence may serve as protective psychological resources, while traditional kinship structures and caregiving roles remain significant. Increased public visibility of LGBTQ+ individuals, through pride events, affirmative media representation, and legal reforms such as the legalization of same-sex marriage, signals a growing institutional recognition. However, structural discrimination and health inequities persist [19,20]. Little research has examined how LGBTQ+ individuals in midlife and older age in Thailand engage in self-care within these evolving yet ambivalent socio-cultural conditions. This study is informed by the frameworks of mental health literacy [21,22], cultural ecology theory [23], and culture–personality theory [24] to explore the intersections of identity, culture, and self-care in later life. By conceptualizing self-care as both an individual practice and a culturally embedded response to systemic challenges, this study seeks to advance a more nuanced understanding of mental health self-care in LGBTQ+ individuals aging in non-Western contexts. Accordingly, the study aims to explore the socio-cultural backgrounds that shape mental health self-care among Thai LGBTQ+ adults in midlife and older age, and to investigate the lived experiences of self-care practices related to mental health within this population.

## Materials and methods

### Design

This study employed a qualitative design with a thematic analysis approach, situated within the constructivist paradigm. The constructivist paradigm assumes that reality is socially constructed and context-dependent, which is particularly salient when exploring the lived experiences of marginalized populations such as LGBTQ+ individuals in midlife and later life [25,26]. This orientation enabled an in-depth examination of how cultural, social, and structural contexts shape individual understandings and practices of mental health self-care. Thematic analysis was used to identify patterns of meaning across participants’ narratives, allowing culturally embedded interpretations of mental health self-care among older LGBTQ+ Thais to emerge inductively and without imposing a predetermined analytical framework.

A qualitative design was chosen to capture participants’ meanings, values, and narratives that may be overlooked in quantitative research. This method allows for a nuanced understanding of identity, intersectionality, and resilience, which are inherently fluid and context-specific [27]. In addition, the use of thematic analysis, as outlined by Braun and Clarke [28], supports the identification of latent and explicit themes across the data, enabling the research to trace patterns of meaning and variation while remaining attentive to power, marginalization, and cultural specificity.

### Participants

Participants were purposively selected based on the following inclusion criteria: (a) being aged 50 years or older, (b) self-identifying as lesbian, gay, bisexual, transgender, queer/questioning, intersex, asexual, or other non-heteronormative identities (LGBTQ+), and (c) expressing willingness and capacity to share personal experiences related to mental health and self-care. Individuals were excluded if they were unable to participate in all stages of the research process, such as data collection and follow-up, or if they voluntarily withdrew from the study at any point. This approach ensured that participants were both relevant to the study aims and able to engage meaningfully in the research process.

A total of 30 participants was determined based on thematic saturation. Saturation was assessed iteratively during data collection and preliminary analysis. After each interview, the research team reviewed emerging codes and preliminary themes to determine whether new concepts, meanings, or patterns continued to emerge. As data collection progressed, recurring patterns became evident, and no substantially new codes or themes emerged that would alter the existing thematic structure. The data were therefore considered sufficiently rich and conceptually developed to support the final themes.

### Data collection

We employed a semi-structured interview guide to facilitate in-depth, context-sensitive exploration of participants’ lived experiences. The guide was developed with careful consideration of cultural appropriateness, sensitivity to gender and sexual diversity, and the emotional nature of mental health topics. All authors are trained in qualitative research and have substantial experience conducting fieldwork with older adults and LGBTQ+ populations. Their familiarity with interpretive inquiry and reflexive interviewing techniques contributed to the depth and ethical rigor of the data collection process.

The interview guide was designed to elicit participants’ experiences, beliefs, and coping strategies related to mental health and self-care. It was informed by prior research on mental health literacy, cultural factors, and LGBTQ+ aging, and was piloted and refined prior to data collection. Example questions from the interview guide include:

1. How would you define “mental health” in your own words?
2. What activities help you feel emotionally well or mentally balanced in daily life?
3. How have your life experiences as an LGBTQ+ person shaped how you care for your mental health?
4. Have you ever sought mental health services? What motivated or prevented you?
5. What role do culture, religion, or family values play in how you view and manage mental health?
6. What kinds of support, formal or informal, do you rely on when you’re stressed or overwhelmed?

The guide also included questions regarding personal background, such as age, profession, educational history, place of origin, and community context. Additionally, participants were asked to reflect on their gender and sexual identity, past experiences with stigma or affirmation, and practices of emotional resilience.

In addition to recorded data, researchers maintained reflexive fieldnotes during and immediately after interviews. These notes captured contextual details, non-verbal observations, and the interviewer’s emotional responses or interpretive reflections, which later informed the analytic process.

The study received ethical approval from the Human Research Ethics Committee of Srinakharinwirot University, Thailand (Protocol No. SWUEC-672321). Participant recruitment took place between 1 October 2024 and 31 October 2024. Data collection was conducted during this period through online semi-structured in-depth interviews.

Participants were recruited through purposive sampling, with the initial outreach conducted via targeted Facebook posts distributed through LGBTQ+ community networks and allied social platforms. Interested individuals were invited to complete a brief online screening questionnaire via Google Forms, which included items on age, gender identity, sexual orientation, and willingness to participate. The aim was to ensure diverse representation across sexual and gender identities as well as age brackets, specifically, 15 participants aged 50–59 and 15 participants aged 60 and above.

To ensure quality and ethical consistency in data collection, interviewers underwent training in qualitative research methods, cultural sensitivity, and LGBTQ+ -affirming communication. Semi-structured, in-depth interviews were conducted online via Zoom to accommodate geographic diversity and minimize travel-related barriers. Video was enabled throughout the interview to allow for observation of participants’ facial expressions and body language, enhancing contextual interpretation.

Each interview session began with (a) a self-introduction by the interviewer, (b) a clear explanation of the research aims and ethical protocols, and (c) a request for permission to audio-record the conversation. Each interview lasted approximately 60–90 minutes. Written informed consent was obtained electronically prior to the interview, and verbal consent was reconfirmed and audio-recorded at the beginning of each interview session. Participants were offered a financial honorarium in recognition of their time, insights, and contributions to the study. All interviews were audio-recorded with participants’ permission and transcribed verbatim for subsequent thematic analysis.

### Analytical strategy

Qualitative data were analyzed using Braun and Clarke’s thematic analysis [28]. Verbatim interview transcripts were reviewed repeatedly to develop a holistic understanding of participants’ narratives. The analysis attended to both semantic and latent levels of meaning. Semantic coding identified participants’ explicit accounts of mental health self-care, while latent interpretation examined the broader cultural meanings underlying these accounts, including Buddhist ethics, kinship obligations, gender norms, and structural inequalities. The semantic themes are presented in the Results, whereas the latent interpretive analysis is developed in the Discussion. An initial round of open coding was conducted to identify recurring patterns, concepts, and significant phrases. These preliminary codes were developed collaboratively during same-day, chat-based debriefings following each interview session, a practice recommended in classic qualitative methodology to enhance analytic credibility and ensure depth while maintaining temporal proximity to the data [29].

Following open coding, the research team engaged in iterative analytic comparison and theme development. Codes were refined, merged, or expanded through multiple team discussions. To strengthen the credibility and trustworthiness of the analysis, peer debriefing and investigator triangulation were employed throughout the process [30]. Discrepant interpretations were discussed and resolved through consensus to ensure the final themes accurately reflected the participants’ lived experiences within their socio-cultural contexts.

### Ethics

This study received ethical approval from the Human Research Ethics Committee of Srinakharinwirot University, Thailand, on June 18, 2024 (Protocol No. SWUEC-672321). All participants provided informed consent before participation, and pseudonyms were used to maintain confidentiality. Particular attention was given to safeguarding participants’ well-being, ensuring affirming interactions, and respecting diverse gender and sexual identities throughout the research process. No deviations from the approved study protocol occurred after ethical approval was obtained.

### Reflexivity and rigor

With academic backgrounds in behavioral and health sciences, we identify as four women from middle-class Thai families and one woman from an Arab background, who have all attained doctoral-level education. These positionalities inevitably inform the lenses through which we approach this research. Our professional training and prior experiences working with older adults and marginalized communities, particularly sexual and gender minorities, have contributed to a heightened sensitivity toward issues of social inequality, aging, and stigma.

Drawing from our lived experiences and academic trajectories, we approach this study through a constructivist paradigm, which recognizes that knowledge is co-constructed through interactions between researchers and participants. This perspective acknowledges that our pre-existing assumptions, social identities, and disciplinary training shape how we engage with participants, analyze narratives, and make meaning from the data. As Thai and Arab women scholars who have been educated both locally and internationally, we are conscious of the cultural assumptions we may bring to the research process, particularly when working with populations whose identities and lived realities may diverge significantly from our own.

Two of the authors have conducted research and fieldwork with LGBTQ+ communities in urban Thailand, focusing on issues related to mental health, aging, and gender. While their experiences are grounded primarily in metropolitan settings, their engagement with participants from diverse provincial, generational, and socioeconomic backgrounds has contributed to a more nuanced understanding of the complex realities faced by LGBTQ+ adults in midlife and older age. These insights have shaped the team’s reflexive approach to fieldwork and analysis, particularly in exploring intersecting identities, structural marginalization, and community-based resilience across varied Thai cultural contexts.

In striving to uphold reflexivity and ethical integrity, we continuously interrogated how our own social locations might shape our engagement with the participants, particularly in terms of power, trust, and interpretation. We adopted open-ended, non-directive interview techniques to allow participants to articulate their experiences without imposing pre-established theoretical frameworks. Moreover, we reflected critically on how language, gender dynamics, and cultural norms might influence what participants chose to disclose and how they framed their own self-care practices. Through these reflexive strategies, we sought to position ourselves not as detached observers but as ethical co-learners, committed to representing the voices and meanings of participants within their specific socio-cultural contexts.

In addition to reflexive practice, multiple strategies were employed to enhance the trustworthiness of the findings. Data triangulation was applied by drawing upon multiple sources, including in-depth interviews and field observations, to strengthen the credibility and richness of the data and to cross-validate emerging themes. Peer debriefing was conducted through independent coding by four researchers. The coding results were then compared and discussed in team meetings, and discrepancies were resolved through consensus to refine the final codes and themes. Furthermore, member checking was undertaken by sharing preliminary themes and categories with five participants, enabling them to verify the accuracy and completeness of the interpretations.

## Results

### Participant characteristics

A total of 30 participants, aged between 50 and 68 years, were included in this study, with a mean age of 57.83 years (SD = 5.30). The participants reflected substantial diversity in terms of both gender identity and sexual orientation. Based on sex assigned at birth, 13 participants were assigned female and 17 were assigned male. Reported gender identities included 2 transgender men, 6 transgender women, 4 non-binary individuals, 1 queer-identifying individual, and 17 cisgender individuals (comprising 9 cisgender men and 8 cisgender women).

In terms of sexual orientation, the participants identified as follows: 15 gay men, 11 lesbian women, 3 bisexual individuals, and 1 asexual individual. This range of sexual orientations and gender identities underscores the heterogeneity of the participants and provides a robust foundation for exploring the intersections of aging, gender, and mental health self-care.

### Thematic findings

Thematic analysis of narratives from 30 participants generated two themes and ten sub-themes that capture both the socio-cultural contexts shaping mental health self-care among Thai LGBTQ+ adults in midlife and older age, and their lived experiences of self-care practices. These findings provide insight into how participants negotiated cultural norms, community expectations, and personal strategies to maintain psychological well-being. To improve clarity, a consistent Theme and Sub-theme labelling system is used throughout the Results section. Table 1 presents an overview of the two themes and ten sub-themes, including short descriptions, representative participant quotations, and saliency indicators showing the extent to which each theme was reflected across participants’ narratives.

**Table 1:** Summary of themes, sub-themes, representative quotations, and saliency.

Theme	Sub-theme	Description	Representative participant quotation	Relative saliency
Theme 1: Socio-cultural backgrounds shaping mental health self-care	1.1 Family and childhood memories	Family structures, gender expectations, kinship roles, and family responsibilities shaped early self-worth, resilience, and later mental health self-care.	“They [parents] saw me as a normal person. They never said anything negative” (P4).	Broad
	1.2 Community environment	Schooling, livelihood trajectories, and community relationships shaped identity formation, belonging, resilience, and coping strategies.	“The teacher said it wasn’t allowed… I had to dress properly. They said I couldn’t be like this” (P17).	Broad
	1.3 Cultural beliefs and mental health	Cultural, Buddhist, and spiritual beliefs shaped coping, meaning-making, self-acceptance, and responses to emotional distress.	“I went to the temple for peace of mind. I like the silence, and I feel that at a certain age, one should begin going to the temple.” (P4).	Broad
	1.4 Intimacy and romantic experience	Romantic, sexual, and caregiving relationships shaped identity, vulnerability, autonomy, emotional survival, and meanings of love.	“I’ve taken care of my boss for 30 years… we don’t need to be lovers. This care itself makes life complete” (P12).	Partial
Theme 2: Lived experiences of mental health self-care	2.1 Coping with stigma and discrimination	Participants coped with stigma through emotional regulation, selective avoidance, boundary-setting, and spiritual coping.	“When we recognize and understand the anger that arises, we can return to our mind more quickly and realize that these things are natural truths: they arise, exist, and eventually fade.” (P28).	Broad
	2.2 Barriers to mental health services	Stigma, misunderstanding, negative encounters, and economic constraints limited access to formal mental health care.	“They think going to see a psychiatrist means you’re insane… I’m still not brave enough to consult” (P1).	Partial
	2.3 Cultivating joy, pride, and self-reliance	Joy, positive thinking, pride, self-reliance, and inner protection supported resilience, dignity, and psychological well-being	“I only do what I can without making myself uncomfortable. I don’t care how much others donate; I only care about how much peace I feel. I am happy simply with my own way of giving.” (P28).	Broad
	2.4 Community contribution and advocacy	Community care, volunteering, digital advocacy, and everyday advocacy transformed personal struggle into collective support and purpose.	*“We never ask each other, ‘What are you? What am I?’… We just are the way we are”* (P5).	Partial
	2.5 Physical health as mental anchor	Exercise, health routines, chronic illness management, caregiving, and holistic self-care supported emotional balance.	“If our health is good… everything we have becomes more valuable. Health must come first.” (P12).	Broad
	2.6 Self-education on mental health	Digital learning, peer knowledge, life reflection, and sharing personal experience strengthened mental health literacy and self-care.	“I listened to Dr. Prawase on YouTube. He said to take care of myself… so I started doing that, and gradually it got better” (P3).	Broad

Saliency key: Broad = reflected across many participants’ narratives; Partial = reflected by a smaller subset of participants. Saliency indicates the relative breadth of endorsement rather than a quantitative frequency count.

### Theme 1: Socio-cultural backgrounds shaping mental health self-care

#### Sub-theme 1.1: Family and childhood memories.

Participants’ narratives revealed that family structures, gender norms, and early socialization played a significant role in shaping their psychological resilience and approaches to mental health self-care. Support, discipline, pressure, and emotional ambivalence within families and kinship structures became both protective and risk factors in their long-term well-being. Within this sub-theme, four key analytic points emerged:

***Family support and upbringing:*** Family acceptance and emotional security were described as critical protective factors in participants’ lives. Supportive parenting particularly unconditional acceptance despite gender nonconformity helped cultivate early self-worth and resilience. In contrast, emotional distance or pressure to conform created long-term psychological challenges. One participant shared: *“They [parents] saw me as a normal person. They never said anything negative”* (P4). In contrast, another participant reflected, *“My father did not understand me. I felt he did not love me… He wanted me to attend the police academy, but I knew I would never succeed there”* (P18).

***Gendered expectations and patriarchal pressure:*** Participants frequently described how rigid gender expectations often rooted in patriarchal family values impacted their emotional development. Fathers were described as emotionally distant and disciplinarian, while mothers tended to reinforce traditional gender roles or submit to paternal authority, as exemplified by this participant: *“I lacked warmth from both father and mother… Father was very masculine… Father never hugged me.”* (P18). These expectations discouraged emotional expression and contributed to feelings of alienation or internal conflict.

***Sibling dynamics and kinship roles:*** In large Thai families, sibling relationships and extended kinship networks acted as both stressors and sources of resilience. Participants reported being compared to siblings, forced into gender-conforming behaviors, or subjected to ridicule. One participant shared: *“My older brother liked to force me to play football and dress like a boy. But I liked wearing skirts and wanted to be a girl since kindergarten.”* (P17). However, in some cases, kin or siblings also offered protection or a sense of belonging. One participant recalled, *“My relatives were friendly and even happy about who I am”* (P1). These ambivalent dynamics deeply shaped participants’ sense of self and mental health practices.

***Burden of responsibility:*** Participants frequently described a lifelong expectation to shoulder responsibility for their families, a burden that shaped their emotional experiences and life trajectories. Rather than receiving care, many felt compelled to provide it, financially, emotionally, or through fulfilling familial duties such as securing housing, caring for aging parents, or maintaining family honor. These expectations often functioned as both pressure and motivation, driving participants to work hard, delay personal aspirations, or suppress aspects of their identity to meet family obligations.

One participant reflected, *“I only thought about how to make my family’s life better… I wanted to quickly buy a house for my parents… Now, the person I loved most, I wanted them to be happy, but they are no longer with me.”* (P14).

#### Sub-theme 1.2: Community environment.

Participants’ life stories reflected the influence of broader Thai socio-cultural and structural contexts on identity formation and mental health self-care. From early schooling to livelihood strategies and everyday interactions with neighbors, participants navigated a world where gender norms, class mobility, and communal expectations shaped their psychological resilience and coping strategies. These experiences illustrate how place, education, and community belonging are not merely background conditions but active components of LGBTQ+ individuals’ mental health trajectories.

***Schooling:*** For many participants, schools were the first social institutions where they became acutely aware of gender expectations and the boundaries of socially acceptable behavior. In Thailand, where gendered school uniforms and moral discipline are institutionalized, schools were both spaces of early trauma and sites of resistance. Participants recounted how enforcement of gender norms through punishment or ridicule deeply affected their sense of self and emotional well-being. One participant described that *“The teacher said it [dressing differently from my assigned sex at birth] wasn’t allowed. They called me in for punishment, squeezed my arm, and shook me… I had to dress properly. They said I couldn’t be like this.”* (P17).

***Socioeconomic mobility and livelihood trajectories:*** Education and employment were interconnected pathways through which participants sought stability, dignity, and recognition. Education provided opportunities for social mobility, particularly for those who viewed academic achievement as a means to uplift their families. As one participant shared, *“I grew up poor in Rayong and all I could think about was how to make life better for my family. I studied hard, became the top student, and earned a scholarship to Chulalongkorn University. That moment was the greatest pride of my life”* (P14).

However, translating educational success into economic security was not guaranteed. Participants’ work experiences revealed both empowerment and vulnerability. For some, employment became a source of purpose and resilience, *“All I thought about was how to make life better for my family… I worked hard because I wanted to buy a house for my* *parents”* (P14). For others, discrimination undermined their ability to remain employed, such as one participant who recalled, *“My boss bullied me by sending me to distant branches until I had to resign”* (P8). These accounts highlight how socioeconomic mobility was shaped not only by personal effort but also by structural barriers tied to gender identity and social expectations.

***Community relationships and belonging:*** Neighborhoods and local communities played a dual role in shaping participants’ emotional landscapes. In rural and peri-urban areas, tight-knit relationships provided social support but also subjected individuals to surveillance, gossip, or even boundary violations. In contrast, some found ways to establish trusting, respectful relationships with neighbors, building informal safety nets essential for aging well. These mixed experiences highlight the significance of localized social networks in Thai society. One participant shared, *“I cooked and shared food with my neighbors… We trusted each other so much that they even kept a spare key to my house.”* (P13).

#### Sub-theme 1.3: Cultural beliefs and mental health.

Cultural and spiritual beliefs played a significant role in shaping the mental health experiences of LGBTQ+ individuals in Thailand, particularly in later life. While religion and cultural practices offered grounding, routine, and meaning in times of personal hardship, they also reinforced silence, guilt, and conformity. Several participants navigated these contradictions by reframing their relationship to tradition embracing certain values like mindfulness or filial piety while rejecting gender-essentialist or heteronormative interpretations. In this way, participants demonstrated cultural negotiation: not full assimilation nor total rejection, but an ongoing reshaping of beliefs to support self-acceptance and mental wellness.

***Evolving gender perceptions across generations:*** Thailand’s cultural history reflects both tolerance and limitation in relation to gender diversity. In earlier generations, rigid binary gender roles, reinforced through family, education, and media, often silenced LGBTQ+ identities, labeling them as “deviant” or “inappropriate.” However, recent decades have seen greater social visibility, especially with more LGBTQ+ representation in media, shifts in public opinion, and legal discourse.

Participants reflected on this transformation, recognizing both the pain of growing up under rigid norms and the relief they feel seeing younger LGBTQ+ people embraced more openly. The shift is bittersweet: a mixture of pride, hope, and unresolved grief for what was once denied. One participant reflected, *“Right now, these LGBT kids are lucky, you know, that they are more accepted in society than we ever were”* (P4). Another echoed this sentiment by noting that acceptance had expanded beyond public discourse and into personal relationships: *“As society becomes more accepting, the people around me also accept me more”* (P29).

***Religious beliefs and spiritual practices:*** Buddhism is a central component of Thai life and moral discourse. For LGBTQ+ participants, Buddhist teachings, particularly those emphasizing impermanence, compassion, and detachment, provided frameworks for resilience and self-reflection. Some participants found comfort through merit-making, meditation, and temple visits, especially when dealing with loss or trauma. Religious rituals were also used to honor parents, fulfill spiritual obligations, or regain emotional balance. However, the interplay between Buddhist morality and LGBTQ+ identities was complex. While Buddhist doctrine does not explicitly condemn homosexuality, certain interpretations (especially around precepts like the Third Sila regarding sexual misconduct) have historically been used to shame or marginalize non-heteronormative identities. Participants responded by selectively adopting teachings that supported peace of mind while rejecting others that caused guilt or self-blame. One participant shared, *“I went to the temple for peace of mind. I like the silence, and I feel that at a certain age, one should begin going to the temple.”* (P4).

***Leisure and well-being practices:*** Beyond formal religion, participants emphasized the importance of daily rituals and leisure activities in promoting mental health. These ranged from spiritual practices like barefoot walking and grounding, to hobbies such as gardening, cooking, and travel. Such practices were often framed not only as enjoyable but essential, allowing for physical health, emotional release, and mental clarity. Participants described how maintaining balance, a core Thai value, guided their self-care. Aging was seen as a time to slow down, enjoy simplicity, and focus inward. For many, these practices helped manage the long-term effects of trauma, stress, and social exclusion without reliance on formal psychological services. One participant explained, *“Grounding helps balance my body, I walk barefoot on the grass, it keeps me healthy and costs nothing.”* (P21).

***Painful memories and traumatic relationships:*** While cultural beliefs offered tools for resilience, they also contributed to environments that allowed psychological wounds to go unaddressed. Participants shared stories of painful relationships, sexual violence, and betrayal, many of which were compounded by shame, silence, and lack of support. Several recalled traumatic experiences involving teachers, monks, or family members, with no clear path to justice or healing due to societal taboos and institutional barriers. In some cases, religion was used to cope or rationalize these events. In others, it deepened feelings of sin or unworthiness. Despite this, many participants worked through these experiences by finding personal meaning, accepting emotional scars, and rebuilding life on their own terms. One participant shared, *“I was raped by a monk… I had to endure it while staying in the temple for three years.”* (P17).

#### Sub-theme 1.4: Intimacy and romantic experience.

For older LGBTQ+ Thais, intimacy was deeply intertwined with their sense of identity, social belonging, and emotional survival. Across their lifespans, romantic and sexual experiences provided spaces for both empowerment and vulnerability. While many participants encountered rejection, heartbreak, or trauma, others recalled warmth, affirmation, and long-term emotional bonds even outside conventional notions of romantic relationships. Some redefined partnerships beyond labels; others embraced singleness as a conscious and liberating choice. Together, these narratives reveal how love was reimagined as care, understanding, and dignity, not just romance or sexual connection.

***Fluid relationships, emotional labor, and autonomy***: Participants’ romantic and sexual experiences evolved across the life course, reflecting shifting meanings of love, intimacy, and self-worth. Early relationships were often sources of exploration, affirmation, and emotional intensity, yet also spaces of heartbreak, violence, or betrayal in a society that stigmatized LGBTQ+ identities. These formative experiences shaped lifelong emotional patterns and coping strategies. As participants aged, many redefined commitments on their own terms, prioritizing peace, self-respect, and mental health over societal expectations of partnership or marriage. For some, chosen families, caregiving bonds, or long-term companionship provided a profound sense of belonging without romantic labels.

One participant explained, *“Being with a woman hurt more… it was both friend and lover in one, far deeper than teenage puppy love”* (P30). Another reflected on non-romantic commitment as a form of emotional fulfillment: *“I’ve taken care of my boss for 30 years… we don’t need to be lovers. This care itself makes life complete”* (P12).

### Theme 2: Lived experiences of mental health self-care

#### Sub-theme 2.1: Coping with stigma and discrimination.

For older LGBTQ+ adults in Thailand, experiences of stigma and discrimination were long-lasting yet transformative. Many had endured ridicule, exclusion, and misunderstanding across different life stages, from childhood classrooms and temples to modern workplaces. Their coping strategies reflected both personal resilience and cultural adaptation, often grounded in Buddhist values of emotional balance, self-control, and compassion. Through such strategies, participants learned to coexist within a heteronormative and hierarchical society that often privileges conformity.

***Facing bullying and emotional regulation:*** Many participants traced their early struggles with stigma back to childhood bullying or mockery from peers and teachers, which shaped their lifelong sense of self-worth. While some internalized shame, others learned to defend themselves and later reframed those experiences as lessons in strength and endurance. Over time, emotional maturity and Buddhist mindfulness enabled them to manage anger and sadness constructively. As one participant explained, *“When we recognize and understand the anger that arises, we can return to our mind more quickly and realize that these things are natural truths: they arise, exist, and eventually fade.”* (P28).

This capacity for emotional regulation reflected a deeply Thai way of coping, cultivating consideration, maintaining calm, and transforming pain into understanding. Participants’ ability to interpret suffering through Buddhist teachings helped them reclaim dignity and reduce the internalized effects of discrimination.

***Selective avoidance and conflict navigation:*** Avoidance, modulation of self-expression, and strategic withdrawal emerged as key emotional survival strategies among participants. In a cultural context where direct confrontation is discouraged, LGBTQ+ elders did not perceive avoidance as passivity but as a deliberate form of boundary-setting, emotional intelligence, and self-preservation. Participants described choosing silence, minimizing visibility, or adjusting their tone, clothing, and behavior to navigate family gatherings, workplaces, and religious spaces. These practices allowed them to maintain social harmony while protecting their psychological well-being in environments where gender diversity remained stigmatized.

As one participant shared, *“I walk away and avoid confrontation. Most of the time it’s just teasing. Don’t use emotions… If something lingers, I’ll clear it up later”* (P22). Another described calibrating visibility as a form of self-protection: *“I will not stand out too much, but I also will not lag behind”* (P12).

***Cultural routines and spiritual coping:*** Daily and religious routines served as powerful emotional anchors. Participants described engaging in merit-making, temple visits, and Buddhist rituals to connect with lost loved ones and maintain inner peace. These practices reveal how spirituality becomes both a coping mechanism and a cultural affirmation. For many, participating in temple life and rituals reinforced their social belonging as “good people”, countering societal perceptions of LGBTQ+ identities as morally deviant. One participant shared, *“My mother passed away in 2021, so I choose to give alms every Monday, the day she died… it feels like she goes along with me.”* (P5).

#### Sub-theme 2.2: Barriers to mental health services.

In Thailand, entrenched myths and sexual prejudice shaped access to mental health care, with many participants reporting they had never sought professional services, relying instead on the internet. Those who did seek care often described negative experiences, such as receiving advice that felt dismissive or irrelevant, which further reinforced distrust and spread misconceptions about mental health services.

***Stigma, rumors, and misunderstanding:*** Stories circulated within communities that seeing a psychiatrist meant being mentally unstable. Participants expressed hesitation to seek help because they feared being judged or labeled, and some noted that transgender identities had historically appeared alongside terms related to mental illness in official documents. Such perceptions shaped how participants understood mental health care and their willingness to access it. One participant shared, *“They think going to see a psychiatrist means you’re insane… I’m still not brave enough to consult.*” (P1).

***Negative encounters:*** For those who sought help, some participants reported distressing interactions that undermined their trust in mental health professionals. Accounts included unethical behaviors, discriminatory remarks, and experiences that left them feeling unsafe and reluctant to return for care. One participant recalled, *“I was sexually harassed by a psychiatrist… I am afraid every time I see a doctor now”* (P18). Such encounters reinforced hesitation to seek formal support and deepened psychological vulnerability.

Others, however, described more positive interactions, where empathetic practitioners created a sense of safety and relief. As one participant noted, *“If I can talk with someone I trust, half of the problem is gone”* (P24).

***Economic constraints:*** Economic limitations shaped participants’ decisions about whether to seek mental health support. Many perceived therapy as costly and unaffordable, making it an option reserved for those with greater financial resources. Even with partial reimbursement, expenses remained burdensome and discouraged continued care. As one participant shared, *“I paid over 8,000 baht, and got 4,000 back from social security… the nurses even sat and talked with me.”* (P13).

#### Sub-theme 2.3: Cultivating joy, pride, and self-reliance.

Daily rituals, creative pursuits, and queer pride narratives fueled psychological resilience, with many participants finding happiness and pride in living self-reliantly. Building confidence and self-worth through independence, community gathering, and social contribution allowed them to strengthen their identities. By joining group activities and engaging in community initiatives, they developed a sense of belonging and cultivated lasting joy and psychological well-being.

***Joy through simplicity and giving:*** Happiness was defined not by material success but by freedom, simplicity, and generosity. Participants linked joy to independence and the act of giving reflects values deeply embedded in Thai culture. As one participant noted, *“I only do what I can without making myself uncomfortable. I don’t care how much others donate; I only care about how much peace I feel. I am happy simply with my own way of giving.”* (P28).

***Positive thinking and emotional resilience:*** Optimism became both a survival mechanism and a moral stance. Participants consciously trained themselves to reinterpret adversity through positive thinking and humor. This perspective illustrates how psychological resilience is intertwined with Buddhist notions of impermanence and acceptance, finding peace through inner control rather than external validation. As one participant shared, *“I am not a stressed person; I am positive. Many people tell me I think positively, and I believe it too.”* (P21).

***Pride and self-reliance as dignity:*** Pride for participants was not arrogance but moral fulfillment from education, work, and contribution. One participant shared, *“I feel proud to have reached the highest level of education, earning a doctorate and using my knowledge to help others.”* (P27).

Such pride affirms LGBTQ+ elders’ social worth in a context where respect is often tied to productivity and filial duty. Self-reliance, likewise, reflected the dignity of not being a burden. As one participant shared, *“We must control ourselves… we cannot always rely on others. We must be strong, not weak-hearted.” (P17)*.

***Immunity and inner protection:*** Repeated experiences of betrayal and loss led to the development of psychological “immunity,” a protective shield balancing caution and self-compassion. This form of inner immunity parallels the Buddhist teaching of equanimity amid suffering showing how maturity transforms emotional scars into wisdom.

*“Now, if anyone invites me to invest, I block them right away… I’ve been deceived before and ruined. I won’t believe easily again.” (P8)*

#### Sub-theme 2.4: Community contribution and advocacy.

Volunteering and organizing were described as healing practices that fostered resilience, dignity, and belonging. By contributing to others, participants strengthened self-worth, affirmed queer pride, and transformed personal struggles into shared empowerment. Acts of helping others reflected deeply rooted Thai values of kind-hearted giving and reciprocal gratitude, while community engagement redefined what it means to live well and grow old as LGBTQ+ in Thailand.

***Communal care and social contribution:*** LGBTQ+ networks provided crucial emotional support, belonging, and identity affirmation. These spaces, including public gatherings and queer cultural events, allowed participants to feel accepted without explanation. As one participant noted, “*We never ask each other, ‘What are you? What am I?’… We just are the way we are*” (P5), illustrating how community ties normalized diversity and eased social pressures.

Care within these networks often became practical and life-sustaining. Participants described offering refuge and emotional support to peers rejected by their families. One recalled helping a friend who was struggling with addiction and AIDS, saying simply, *“I told him to come stay with me”* (P18), highlighting how chosen families served as safety nets.

Beyond personal networks, many engaged in acts of public service that blended social contribution with self-worth. Some used personal skills to support others, for instance, one participant *“used [his] hair-cutting skills as free community service”* (P28), while another shared that they *“…organized a charity concert to raise funds for the gynecological cancer institute”* (P9). These efforts transformed care into a meaningful practice that reinforced dignity, purpose, and connection.

***Digital and everyday advocacy:*** Digital spaces such as Facebook and TikTok offered new avenues for connection, expression, and activism. While some participants found online communication emotionally distant, others embraced it as a supportive platform for mental well-being, education, and empowerment. Personal storytelling and online volunteering allowed them to reach wider audiences, especially in a culture where open confrontation is often discouraged. One participant noted, *“…online communities bring people together and support mental well-being.” (P3)*.

At the same time, participants practiced what might be called “quiet advocacy” in everyday life, living authentically, mentoring youth, and speaking calmly about gender diversity. One participant shared, *“Today, society is more open and accepting… many are so talented, sometimes more than straight men and women, which makes the world recognize them.”* (P29).

Through such subtle, relationship-based change, LGBTQ+ elders contributed to a gradual cultural shift toward inclusion and respect.

#### Sub-theme 2.5: Physical health as mental anchor.

Attention to exercise, nutrition, and chronic illness management supported emotional stability. Older LGBTQ+ adults described health care as a holistic practice combining physical activity, creative engagement, balanced routines, and mental well-being to cope with life pressures and sustain resilience.

***Holistic self-care and emotional balance:*** Participants engaged in physical activity, walking, swimming, dancing, or gym workouts, not merely for fitness but for emotional renewal. Exercise and mindfulness served as parallel routes to regulating stress and restoring joy. Many viewed their daily health routines as sacred acts that linked self-discipline with compassion. Such practices reveal a holistic concept of wellness, where physical vitality, spiritual composure, and emotional clarity reinforce each other. One participant shared, *“If our health is good… everything we have becomes more valuable. Health must come first.”* (P12).

***Connection and caregiving as emotional grounding:*** Caring for others, elders, relatives, friends, or community members, was described as both a responsibility and a source of happiness. These caregiving roles reflected the Thai cultural expectation of reciprocal care while giving LGBTQ+ elders a renewed sense of social belonging. Such acts of love and service transformed conventional images of dependency in old age into empowerment through nurturing others. As one participant explained, *“My pride is being the one who stays with my mother, caring for her and my aunt, becoming the center of the family.” (P28)*.

#### Sub-theme 2.6: Self-education on mental health.

Continuous learning was central to participants’ mental self-care. Many turned to digital media, peers, and life reflection to expand mental-health literacy, combining Buddhist insights with modern psychological ideas. Knowledge was viewed not merely as information but as moral cultivation.

***Digital and experiential learning:*** Participants frequently relied on YouTube, Facebook, and Google to learn about emotional regulation, mindfulness, and LGBTQ+ rights. They also drew upon peers’ life stories and community discussions as informal educational resources. One participant described how online guidance directly informed self-care practices, stating, *“I listened to Dr. Prawase on YouTube. He said to take care of myself… so I started doing that, and gradually it got better”* (P3). Another participant explained that they not only consumed information but also shared it with others, particularly health-related resources, noting, *“I sent links and show friends how to use Google to search for information”* (P22). This blend of digital access and peer-based learning demonstrates how Thai LGBTQ+ elders creatively integrate new knowledge into traditional ways of knowing.

***Applying and sharing knowledge for growth:*** Participants applied insights from self-learning to everyday life, practicing mindfulness, setting boundaries, and accepting themselves with compassion. Many extended this learning to others, teaching meditation, giving online talks, or mentoring peers through personal experience.

One participant explained, *“Bad experiences can harm your mental health, but if you adapt quickly, they can also give you the strength to carry on.”* (P30). And another shared,

*“I once attempted suicide… but now I can return to society and serve as a role model for others with mental health struggles.”* (P19).

Sharing became both altruism and affirmation, a way to turn pain into wisdom and reinforce the belief that resilience is learnable and collective.

## Discussion

This study illuminates the socio-cultural and structural dimensions shaping the mental health self-care of aging LGBTQ+ adults in Thailand. Rather than being a purely individual process, self-care emerged as a socially situated practice deeply embedded in family relationships, cultural values, religious beliefs, and community networks. The findings highlight that self-care must be understood through the lens of the social determinants of health [31], which recognize that well-being is produced through the interaction of personal agency and broader social, economic, and cultural structures. In this context, participants’ lives reflected how social marginalization, limited welfare access, and cultural scripts surrounding gender and family intersect to shape both vulnerability and resilience in later life.

Consistent with Minority Stress Theory [3], participants’ narratives illustrated the lifelong accumulation of stressors resulting from stigma, discrimination, and social exclusion. Many recalled experiences of ridicule and punishment in childhood for gender nonconformity, institutional rejection in schools and workplaces, and persistent societal prejudice that continued into older age. These experiences mirror cumulative disadvantage models [1], showing how chronic exposure to stigma manifests not only in psychological distress but also in avoidance, internalized shame, and cautious self-regulation. However, within the Thai cultural context, participants reframed endurance and restraint as emotional wisdom rather than weakness. Through practices of calm and mindfulness, they transformed suffering into understanding and maintained social harmony, a form of adaptive coping shaped by collectivist ethics. This culturally grounded response demonstrates that resilience among LGBTQ+ Thais is not merely resistance against oppression, but an ongoing negotiation of dignity within hierarchical and relational social orders.

The findings also resonate with Cultural Ecology Theory [23] and Culture–Personality Theory [24], which posit that human behavior is a product of continuous adaptation between individual dispositions and cultural environments. For older LGBTQ+ adults, Thai Buddhism provided both a source of comfort and a framework for moral reflection. Practices such as meditation, merit-making, and temple participation offered spiritual grounding and a sense of continuity, especially during grief or loneliness. Yet, religious and cultural norms surrounding gender conformity often reinforced silence and guilt, producing an ambivalent relationship between faith and self-acceptance. Participants navigated this ambivalence by selectively integrating traditional values, embracing compassion and detachment while rejecting patriarchal interpretations that invalidate gender diversity [32]. Such negotiation exemplifies a process of cultural re-appropriation, where individuals reinterpret existing moral systems to sustain psychological balance and moral worth.

From a mental health literacy perspective [21,22], participants actively sought knowledge and coping resources through digital media, peer discussions, and self-reflection. Many learned about emotional regulation, mindfulness, and LGBTQ+ rights through YouTube and social media, transforming informal learning into collective knowledge-sharing. These self-education efforts reveal how digital access can compensate for institutional gaps in formal care, particularly in contexts where mental illness remains stigmatized [16,17]. However, myths equating psychiatric consultation with “madness” continued to deter help-seeking. Some participants experienced dismissive or discriminatory encounters with healthcare providers, underscoring systemic inequities within Thailand’s mental health system [20]. The persistence of such barriers illustrates that literacy alone cannot overcome structural exclusion; institutional reforms that address provider bias, affordability, and service accessibility remain essential.

Beyond informational gaps, systemic and cultural forces further constrained help-seeking behaviors. This reluctance stemmed from social stigma that equates psychiatric consultation with “madness” or severe crisis, compounded by financial barriers that rendered therapy inaccessible or perceived as unnecessary. These narratives illustrate how mental health stigma intersects with sexual stigma, reinforcing silence among LGBTQ+ elders who already face social invisibility. The historical classification of transgender identities as forms of mental illness in official documents has also contributed to persistent distrust toward mental health professionals.

The results further affirm that health behaviors are profoundly shaped by social determinants of health, encompassing material conditions, interpersonal relationships, and cultural expectations [33]. At the interpersonal level, kinship obligations and caregiving roles, central to Thai moral life, served as both sources of meaning and emotional strain. Participants derived pride from caring for parents, partners, or community members, transforming care into a moral and spiritual practice of reciprocity. At the community level, religious spaces and neighborhood networks alternated between inclusion and surveillance: temples and local gatherings fostered belonging yet imposed moral boundaries. These ambivalent environments required emotional navigation strategies that balanced authenticity with acceptance, a distinctly Thai mode of resilience rooted in relational ethics. At the structural level, gaps in social welfare, healthcare accessibility, and legal protection continued to marginalize aging LGBTQ+ people, reinforcing the need for policies that integrate equity across age, sexuality, and health systems [34].

Amid these constraints, participants cultivated joy and pride through resilient reinterpretation of self-worth. Their emphasis on self-reliance, volunteering, and mentoring illustrated a transformation from coping to contribution. Acts of caregiving, community engagement, and “quiet advocacy” became mechanisms of healing that reaffirmed their humanity and dignity. These practices align with the Buddhist ethic of merit-making [35] while simultaneously embodying social activism rooted in empathy rather than confrontation. This culturally specific resilience mirrors what Orel and Fruhauf [36] describe as “resilience through reciprocity,” where well-being in later life is sustained by helping others and sustaining moral connection. By transforming pain into altruism, participants transcended victimhood and reframed aging as a process of continued giving and self-realization.

Taken together, these findings suggest that mental health self-care among older LGBTQ+ Thais is situated at the intersection of stress, adaptation, and cultural meaning-making. Psychological endurance was nurtured not only through internal strengths but also through relational, moral, and spiritual resources embedded in Thai society. The integration of multiple theoretical perspectives, Minority Stress, Cultural Ecology, Culture–Personality, and Mental Health Literacy, provides a comprehensive lens for understanding how identity, culture, and structure interact in shaping well-being. The results underscore that resilience is relationally constructed, culturally negotiated, and politically constrained. Therefore, interventions should move beyond individual psychotherapy to address structural and cultural determinants, including inclusive service delivery, community-led support systems, and culturally grounded mental health education.

Ultimately, promoting mental health equity for LGBTQ+ elders in Thailand requires recognizing that self-care is both a personal right and a social responsibility. It must be supported by compassionate policy, inclusive healthcare systems, and sustained public education that values diversity as part of collective moral growth. By centering the lived experiences of older LGBTQ+ Thais, this study contributes to a growing body of global research on queer aging and demonstrates that mental health resilience is not only about survival, but about transforming suffering into wisdom, dignity, and social contribution.

### Strengths and limitations and future research

This study offers one of the few qualitative inquiries exploring mental health self-care among aging LGBTQ+ adults in Thailand, contributing culturally grounded insights to a field still dominated by Western perspectives. Its strengths lie in the use of in-depth, reflexive interviews conducted in participants’ native language, enabling nuanced understanding of emotional, cultural, and structural dimensions of resilience. The integration of multiple theoretical lenses, including Minority Stress Theory, Cultural Ecology, Culture–Personality Theory, and Mental Health Literacy, allowed for a multidimensional interpretation that connects individual agency with broader social determinants of health.

However, several limitations should be noted. The purposive recruitment approach, primarily through Facebook and urban LGBTQ+ networks, may have resulted in a participant that was more digitally literate, technologically connected, and community-engaged than the broader population of older LGBTQ+ adults in Thailand. This may have contributed to the prominence of themes related to digital advocacy, peer learning, community contribution, and online self-education. The findings may therefore less fully reflect the experiences of LGBTQ+ older adults who live in rural areas, experience poverty or social isolation, or have limited access to digital technologies. As a qualitative study, the findings are not intended for statistical generalization, and reliance on self-reported data may introduce recall or social desirability bias.

Future research should extend these findings through mixed-method or longitudinal designs to examine how mental health self-care evolves across life stages and changing policy landscapes. Comparative studies across different Asian contexts would also illuminate cultural variability in resilience processes. Finally, participatory and community-based research could strengthen advocacy and inform culturally attuned interventions to promote mental health equity for LGBTQ+ aging populations.

## Conclusion

Gaining a comprehensive understanding of mental health self-care among middle-aged and older LGBTQ+ adults in Thailand provides critical insights into how personal resilience is embedded within cultural, familial, and structural realities. Self-care for this population is not a set of isolated coping behaviors but an adaptive, meaning-making process that unfolds amid long-term exposure to stigma, discrimination, and social invisibility. Participants demonstrated how Buddhist values, kinship obligations, and community support systems shaped their mental wellness, reflecting the interplay between individual agency and collective resilience. These findings underscore that mental health self-care in aging LGBTQ+ Thais is deeply tied to the social determinants of health, including income stability, social inclusion, healthcare accessibility, and intergenerational support.

To effectively promote well-being and equity among older LGBTQ+ populations, interventions must integrate both psychological and social dimensions. This includes developing culturally attuned, LGBTQ + -affirming mental health services, fostering community-based peer networks, and training healthcare professionals in inclusive and compassionate care. Policies should address structural inequities through social welfare programs, anti-discrimination laws, and health literacy initiatives that empower individuals to seek help without fear or stigma. Collaborative efforts among psychologists, social workers, policymakers, and community leaders can strengthen sustainable support systems for queer aging. By situating resilience within the broader cultural ecology of Thailand, this study highlights the necessity of multidimensional, justice-oriented approaches that link self-care to social transformation and collective dignity.

## Supporting information

S1 Checklist Standards for Reporting Qualitative Research (SRQR). This file contains the completed SRQR checklist, including reporting items, manuscript sections, and corresponding page numbers.
(DOCX)

S2 Checklist PLOS Inclusivity in Global Research Questionnaire. This file contains the completed PLOS Inclusivity in Global Research Questionnaire for this human subject’s qualitative study.
(DOCX)
