# Supplementary material for: Navigating resilience: Mental health self-care among middle-aged and older LGBTQ+ adults in Thailand
Source: PLoS One. 2026 Jul 17;21(7):e0353372. doi: 10.1371/journal.pone.0353372 (PMC13378992; doi:10.1371/journal.pone.0353372)
Supplement: S1 Checklist — This file contains the completed SRQR checklist, including reporting items, manuscript sections, and corresponding page numbers. (DOCX) [file pone.0353372.s001.docx]

**Standards for Reporting Qualitative Research (SRQR)**

**Title:** Navigating Resilience: Mental Health Self-Care Among Middle-Aged and Older LGBTQ+ Adults in Thailand

| **No.** | **Topic** | **Item** | **Page no.** |
| --- | --- | --- | --- |
| S1 | Title | The title clearly identifies the study as a qualitative research project involving LGBTQ+ older adults in Thailand and mentions the use of thematic analysis. | 1 |
| S2 | Abstract | The abstract provides a concise summary of the study’s background, purpose, methodology (qualitative interviews), major findings, and conclusions. | 1-2 |
|  | **Introduction** | |  |
| S3 | Problem formulation | The introduction outlines the lack of non-Western perspectives in mental health self-care research, emphasizing the need to understand how older LGBTQ+ individuals in Thailand manage mental health. | 2-4 |
| S4 | Purpose or research question | The manuscript clearly states the objective of exploring how older LGBTQ+ adults in Thailand engage in mental health self-care within their socio-cultural contexts. | 5 |
|  | **Methods** | |  |
| S5 | Qualitative approach and research paradigm | The study employs a thematic analysis approach and adopts a constructivist paradigm to explore subjective experiences and culturally grounded meanings. | 5-8 |
| S6 | Researcher characteristics and reflexivity | The manuscript discusses the researchers’ positionality, their connection to LGBTQ+ advocacy, and how their backgrounds may have influenced data collection and interpretation. | 10-11 |
| S7 | Context | The study situates itself within Thailand’s cultural, religious, and social contexts, highlighting how these influence participants’ self-care experiences. | 5-6 |
| S8 | Sampling strategy | Participants were selected using purposive sampling to reflect diverse LGBTQ+ identities, regions, and socio-economic backgrounds. Sampling continued until thematic saturation was achieved. | 6-8 |
| S9 | Ethical issues | Ethical approval was obtained from Srinakharinwirot University. All participants gave informed consent. Confidentiality and emotional safety were prioritized. | 9 |
| S10 | Data collection methods | Data were collected through semi-structured, in-depth interviews conducted online. The interview process was flexible to accommodate participant comfort and availability. | 6-8 |
| S11 | Data collection instruments and technologies | An interview guide was used and adapted as needed. Interviews were conducted via Zoom and audio-recorded with participant consent. | 6-8 |
| S12 | Units of study | Thirty LGBTQ+ individuals aged 50–68 participated in the study. Demographic information (e.g., age, gender identity) is summarized in the methods section | 11-12 |
| S13 | Data processing | Interviews were transcribed verbatim. Data were anonymized through the use of pseudonyms and securely stored for analysis. | 8-9 |
| S14 | Data analysis | Thematic analysis was conducted through iterative coding and constant comparison. A codebook was developed collaboratively by the research team. | 8-9 |
| S15 | Techniques to enhance trustworthiness | Credibility was supported through member checking, team debriefings, and triangulation. Reflexive journaling was also used. | 11 |
|  | **Results/findings** | |  |
| S16 | Synthesis and interpretation | Findings are presented thematically, with each theme contextualized within cultural and structural dimensions relevant to Thai LGBTQ+ elders. | 11-31 |
| S17 | Links to empirical data | Direct quotations from participants are included to support and illustrate the analytic themes. | 11-31 |
|  | **Discussion** | |  |
| S18 | Integration with prior work | Results are compared with existing literature on LGBTQ+ mental health and self-care practices, especially in non-Western contexts. | 31-35 |
| S19 | Limitations | The study acknowledges limitations related to sample diversity, online interviews, and generalizability beyond the Thai context. | 35-36 |
|  | **Other** | |  |
| S20 | Conflicts of interest | Authors declare no conflicts of interest. Reflexive consideration of researcher influence is integrated into the discussion. | Online submission form |
| S21 | Funding | Funding information has been provided only in the Funding Statement section of the online submission form, in accordance with PLOS ONE requirements. Funding-related text has been removed from the manuscript. | Online submission form |
