# Supplementary material for: Navigating resilience: Mental health self-care among middle-aged and older LGBTQ+ adults in Thailand
Source: PLoS One. 2026 Jul 17;21(7):e0353372. doi: 10.1371/journal.pone.0353372 (PMC13378992; doi:10.1371/journal.pone.0353372)
Supplement: S2 Checklist — This file contains the completed PLOS Inclusivity in Global Research Questionnaire for this human subject’s qualitative study. (DOCX) [file pone.0353372.s002.docx]

Inclusivity in global research

PLOS’ policy on inclusivity in global research aims to improve transparency in the reporting of research performed outside of researchers’ own country or community and ensures that PLOS publications reporting global research adhere to high standards for research ethics and authorship. Authors of relevant research articles may be asked to complete the questionnaire below, which outlines ethical, cultural, and scientific considerations specific to inclusivity in global research. This questionnaire may be requested when researchers have travelled to a different country to conduct research, if research uses samples collected in another country, research with Indigenous populations or their lands, or if research is on cultural artefacts. Researchers travelling to another country solely to use laboratory equipment will not normally be required to complete the questionnaire. However, the questionnaire can be requested at the journal’s discretion for any submission – if you have been requested to complete this questionnaire by the PLOS journal you submitted to, please do so.

Please complete the questionnaire below and include this as a Supporting Information file with your manuscript. Note that if your paper is accepted for publication, this checklist will be published with your article in the supporting information files. Please ensure that you reference the checklist in the main body of your manuscript. We suggest adding a subsection ‘Inclusivity in global research’ to your Methods section and adding the following sentence: “Additional information regarding the ethical, cultural, and scientific considerations specific to inclusivity in global research is included in the Supporting Information (SX Checklist)”

The questions have been designed to be applicable to a wide range of study types, and there are subsections for both human subjects research and non-human subjects research. If any of the questions are not relevant to your research please mark them as “N/A” as appropriate.

**Ethical considerations, permits and authorship**

*This section is applicable to all research types.*

Provide details as to who granted permissions and/or consent for the study to take place in the Methods section of your manuscript. This should include the names of **all** ethics boards, governmental organizations, community leaders or other bodies that provided approval for the study. If individuals provided approval refer to these people by their role or title but do not list their name(s).

Reported on page number: 9

If there were any deviations from the study protocol after approval was obtained please provide details of these changes in the Methods section of your manuscript.
Did this study involve local collaborators that are residents of the country where the research was conducted or members of the community studied? If you do not have any authors from said communities, please provide an explanation for this below.

Reported on page number: 9

Yes. The study involved local collaborators who are residents of Thailand, where the research was conducted. The research team included Thai researchers with expertise in behavioral science, mental health, aging, and LGBTQ+ community-related research. Local collaborators contributed to study design, recruitment, data collection, analysis, interpretation, and manuscript preparation.

Everyone listed as an author should meet PLOS’ criteria for authorship and all individuals who meet these criteria should be included in the author byline, rather than the acknowledgements. For further information please see the journal’s Authorship Policy.

**Human subjects research (e.g. health research, medical research, cross-cultural psychology)**

Did you obtain written informed consent from a representative of the local community or region before the research took place? How did you establish who speaks for the community? Details of written informed consent obtained from study participants should be reported separately in the Methods section of your manuscript.

No. We did not obtain written informed consent from a representative of the local community or region before the research took place, as the study involved individual survey participation rather than community-level representation or intervention. There was no single formal representative who could speak on behalf of the diverse LGBTQIA+ middle-aged and older adult community in Thailand. Therefore, ethical approval was obtained from the relevant institutional ethics committee, and informed consent was obtained directly from each individual participant before data collection.

How did members of the local community provide input on the aims of the research investigation, its methodology, and its anticipated outcome(s)?

Members of the local community were involved as individual research participants rather than as formal advisors in the study design. They did not provide formal input on the aims, methodology, or anticipated outcomes before data collection. The aims and methodology were developed by the research team based on existing literature, the Thai sociocultural context, and prior research experience with mental health literacy and LGBTQIA+ populations. Community members contributed through their survey responses, which formed the empirical basis of the study findings. We acknowledge that future research would benefit from more direct community consultation during the study design stage.

When engaging with the local community, how did you ensure that the informed consent documents and other materials could be understood by local stakeholders?

The study did not involve formal engagement with a community representative or community advisory group. However, all informed consent documents and study materials provided to participants were prepared in Thai, using clear and accessible language appropriate for local participants. The consent form explained the study purpose, procedures, voluntary participation, confidentiality, potential risks and benefits, and the right to withdraw from the study. Participants were given the opportunity to review the information and ask questions before providing consent. The study documents were also reviewed and approved by the Human Research Ethics Committee of Srinakharinwirot University before data collection.

Will the findings of the research be made available in an understandable format to stakeholders in the community where the study was conducted (e.g. via a presentation, summary report, copies of publications, etc.)? Please provide details of how this will be achieved.

Yes. After publication, the research team plans to prepare a brief Thai-language summary of the key findings using clear, non-technical language. This summary may be shared with relevant academic, health, mental health, aging, and LGBTQIA+ related networks, as appropriate. Copies of the publication or a summary report may also be made available upon request. The dissemination will focus on practical implications for improving mental health literacy and supporting LGBTQIA+ middle-aged and older adults in Thailand.

**Non-human subjects research using specimens/ animals collected as part of the study, or those housed in archival collections. Examples include archaeology, paleontology, botany and zoology.**

Did the permission you obtained from a local authority to perform the study include an agreement on access to outputs and benefit sharing? This may include procedures to enable fair distribution of the benefits and resources arising from the research performed. Please include any details of Prior Informed Consent and Benefit Sharing Agreements obtained. These may be required by field-specific regulations, for example the Convention on Biological Diversity (CBD) and the associated Nagoya Protocol.

Not applicable. This study did not involve non-human subjects, specimens, animals, biological materials, or archival collections.

If the material used in your study was imported, please A) provide the year it was imported and B) indicate whether permits were obtained to import/export the materials used, C) provide details of any permits obtained. If this information is not available, please indicate this.

Not applicable. No materials or specimens were imported for this study.

If you used archival specimens, please state how the material used in your study was acquired by the institute it is held in and provide details of any permits obtained for the original excavations/ sample collection. If this information is not available, please indicate this.

Not applicable. This study did not use archival specimens or materials from archival collections.

How was the potential cultural significance of the materials collected in your study to local communities considered in your research design? Were Indigenous peoples and/or local researchers and institutions involved with archaeological excavations / collection of specimens? If so, please provide a description of their involvement.

Not applicable. This study did not involve the collection of archaeological materials, specimens, biological materials, or culturally significant materials.

If your manuscript includes photographs of human remains please indicate whether authors obtained permission from descendants or affiliated cultural communities to do so.

Not applicable.
